# The Influence of Demographics and Vascular Risk Factors on Glymphatic Function Measured by Diffusion Along Perivascular Space

**DOI:** 10.3389/fnagi.2021.693787

**Published:** 2021-07-19

**Authors:** Yao Zhang, Ruiting Zhang, Yongquan Ye, Shuyue Wang, Yeerfan Jiaerken, Hui Hong, Kaicheng Li, Qingze Zeng, Xiao Luo, Xiaopei Xu, Xinfeng Yu, Xiao Wu, Wenke Yu, Minming Zhang, Peiyu Huang

**Affiliations:** ^1^Department of Radiology, The Second Affiliated Hospital, Zhejiang University School of Medicine, Hangzhou, China; ^2^UIH America, Inc. Houston, TX, United States

**Keywords:** glymphatic system, diffusion tensor imaging, perivascular space, neuroimaging, cerebral vascular disease

## Abstract

Assessing glymphatic function using in-vivo imaging method is of great value for understanding its contribution to major brain diseases. In the present study, we aim to validate the association between a variety of risk factors and a potential index of glymphatic function—Diffusion Tensor Image Analysis Along the Perivascular Space (ALPS index). We enrolled 142 subjects from communities and performed multi-modality magnetic resonance imaging scans. The ALPS index was calculated from diffusion tensor imaging data, and its associations with demographic factors, vascular factors were investigated using regression analyses. We found that the ALPS index was negatively associated with age (β = −0.284, *p* < 0.001). Compared to males, females had significantly higher ALPS index (β = −0.243, *p* = 0.001). Hypertensive subjects had significantly lower ALPS index compared to non-hypertensive subjects (β = −0.189, *p* = 0.013). Furthermore, venous disruption could decrease ALPS index (β = −0.215, *p* = 0.003). In general, our results are in consistent with previous conceptions and results from animal studies about the pathophysiology of glymphatic dysfunction. Future studies utilizing this method should consider introducing the above-mentioned factors as important covariates.

## Introduction

As dysfunction of the glymphatic system is associated with a variety of neuropsychiatric diseases (Jessen et al., 2015; Li et al., 2020; Nedergaard and Goldman, 2020; Huang et al., 2021b), assessing glymphatic function is desired in clinical studies. In recent years, the dilation of perivascular space (PVS) has been widely used to reflect glymphatic dysfunction in clinical neuroimaging studies (Taoka and Naganawa, 2020). However, as its pathological underpinning is complicated and controversial, the precise influence of PVS dilation on cerebrospinal fluid (CSF)/interstitial fluid (ISF) drainage still needs more research to verify (Wardlaw et al., 2020). To address this issue, several in-vivo imaging methods have been proposed. Repeated contrast enhanced magnetic resonance imaging based on intrathecal (Ringstad et al., 2017) or intravenous (Taoka and Naganawa, 2018, 2021) injection of Gadolinium could reflect glymphatic clearance, but the method is invasive and requires multiple brain scans.

Because the foundation of glymphatic system is the motion of CSF and ISF, diffusion tensor imaging may help to reveal the process. Recently, Taoka et al. (2017) proposed a non-invasive method called “Diffusion Tensor Image Analysis Along the Perivascular Space” (ALPS). Two follow-up studies (Yokota et al., 2019; Hsiu-Ling et al., 2021) found decreased ALPS index in patients with Idiopathic Normal Pressure Hydrocephalus (NPH), which was believed a disease with impaired glymphatic clearance. Furthermore, high accuracy could be achieved for diagnosing NPH using ALPS. Compared to normal controls, patients with Parkinson’s disease and cognitive impairment had decreased ALPS (Chen et al., 2020), which was also was negatively correlated with plasma nuclear DNA, mitochondrial DNA levels, and cognitive scores. The association between ALPS and subcortical iron accumulation has also been reported (Zhou W. et al., 2020).

Due to the scarcity of studies employing this technique, the ALPS index still needs validation in clinical imaging studies. Specifically, there is no report of the influence of several important risk factors, including sex, hypertension, and sleep on the ALPS index. Furthermore, the ALPS is hypothesized to reflect glymphatic function around deep medullary veins (DMVs), which may degenerate and become tortuous during aging (Shaaban et al., 2017). Venous degeneration may cause significant influence on the measurement of ALPS, and the scale of its effect should be demonstrated to provide reference for future studies.

In the present study, we aim to investigate the influence of demographic characteristics, vascular risk factors and venous disruption on glymphatic function measured by ALPS in a community cohort. As animal studies have demonstrated that risk factors such as aging, hypertension and diabetes could cause decreased glymphatic clearance, we expect to replicate these findings using this in-vivo imaging method. Moreover, as cerebral small vessel disease (CSVD) is believed related to glymphatic dysfunction, we also investigated the association between CSVD imaging markers and the ALPS index.

## Materials and Methods

### Subjects

We enrolled 142 normal elderly subjects from a prospectively collected dataset. All subjects were recruited through advertisement in communities. The inclusion criteria were: (1) age > 50; (2) with complete multi-modality MRI data. The exclusion criteria were: (1) history of brain trauma, neurological or psychiatric diseases; (2) abnormal brain MRI findings such as hemorrhage, infarction (lacunes were allowed) and other space-occupying lesions. The Pittsburgh Sleep Quality Index (PSQI) was used to measure the sleep condition of the subjects.

All subjects went through a complete assessment of neuropsychiatric conditions, and multi-sequence MRI scans. Hypertension was defined as the presence of any of the following: systolic blood pressure ≥ 140mmHg or diastolic pressure ≥ 90mmHg measured twice in quiet conditions or having self-reported history of hypertension. Diabetes mellitus was defined as the presence any of the following: fasting serum glucose > 7.0mmol/L or postprandial 2h plasma glucose > 11.1mmol/L or having previous history of diabetes. Hyperlipidemia was defined as having elevated level of triglyceride, or total cholesterol, or low-density lipoprotein.

### MR Scan Parameters

All the MR images were acquired using a United Imaging MR790 3.0T scanner (Shanghai, China). T1 weighted images were acquired with a 3D fast spoiled gradient-echo sequence, the parameters were: TR = 6.9 ms, TE = 2.9 ms, flip angle = 9°, Inversion time (TI) = 1,000 ms, field of view = 256^∗^240 mm, voxel size = 1^∗^1^∗^1 mm, 208 sagittal slices. T2 weight images were acquired with a MATRIX (modulated flip angle technique in refocused imaging with extended echo train) sequence, the parameters were: TR = 3,000 ms, TE = 405.46 ms, echo train length = 180, field of view = 256^∗^240 mm, voxel size = 0.8^∗^0.8^∗^0.8 mm, 208 sagittal slices. T2 FLAIR images were acquired with inversion recovery MATRIX sequence, the parameters were: TR = 6,500 ms, TE = 432.48 ms, echo train length = 220, bandwidth = 600 Hz/pixel, field of view = 256^∗^220 mm, voxel size = 1^∗^1^∗^1 mm, 170 sagittal slices. Diffusion tensor images was acquired with an echo planar imaging sequence, the parameters were: TR = 8,682 ms, TE = 75.8 ms, field of view = 224^∗^224 mm, voxel size = 2^∗^2^∗^2 mm, 70 axial slices, diffusion direction = 30, *b* = 0, 1,000, 2,000, phase encoding direction = posterior-anterior. Another b0 image with opposite phase encoding direction was acquired to correct EPI distortions. The acquisition planes were adjusted so that the axial slices were aligned with the AC-PC line, and the brain was in a relatively standard position. Susceptibility weight imaging (SWI) was acquired using a 3D gradient echo sequence, the parameters were: TR = 35.7 ms, first TE = 3.1 ms, last TE = 30.4 ms, number of echo = 8, field of view = 240^∗^240 mm, voxel size = 0.75^∗^0.75^∗^2 mm, 60 axial slices. Several other sequences were acquired, and the total scan time was about 1 h.

### Image Analysis

#### DTI Processing and ALPS Calculation

The processing of DTI was based on FSL 6.0^1^ and MRtrix3^2^. Diffusion images acquired with *b* = 2,000 were removed from analysis, as previous studies showed that ALPS-index had better sensitivity when using *b* = 1,000 images (Taoka et al., 2017). DTI preprocessing included denoising, removing Gibbs artifact, EPI distortion correction and eddy current correction. DTI reconstruction was performed using the diffusion toolbox in FSL. Eigenvector maps and tensor maps were saved for further analysis. Then, all the results were normalized to the MNI space using linear registration (2 mm isotropic voxel size). Vector images were registered using the vecreg command to preserve orientation information.

The goal of the ALPS index is to measure water diffusivity along the direction of the peri-venous space. The basic assumptions are: (1) water diffusion measured in WM near the ventricle body is composed of diffusion from both peri-venous space and WM fiber tracts. (2) As both medulla veins and fiber tracts are running at specific directions, diffusion from the two compartments could be separated by carefully selecting the region of interest (ROI) and direction of measuring.

Several prerequisites should be met for accurate measuring. Firstly, the ROIs should be placed at the level of the upper corner of lateral ventricle body, where the DMVs (as well as peri-venous space) run perpendicular to the ventricular wall (left-right direction in standard space, x-axis). Secondly, the ROIs should be located within the project fiber and association fiber, where the diffusivity was large along the fiber directions but restricted on other directions. The final ALPS index was calculated by the equation (Taoka et al., 2017):

(1) $$A\,L\,P\,S\,i\,n\,d\,e\,x=\frac{\mathrm{mean}\,\left(D\,x\,\_\,p\,r\,o\,j,D\,x\,\_\,a\,s\,s\,o\,c\right)}{\mathrm{mean}\,\left(\mathrm{Dy}\,\_\,\mathrm{proj},D\,z\,\_\,a\,s\,s\,o\,c\right)}$$

**FIGURE 1:**
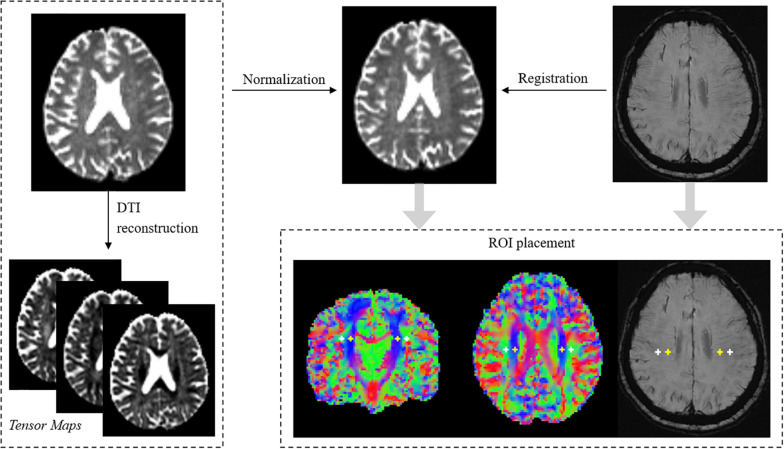
Demonstration of processing pipelines and ROI placement. The locations of ROIs were confirmed on both diffusion and susceptibility images. White crosshairs indicate ROIs in bilateral association fibers. Yellow crosshairs indicate ROIs in bilateral projection fibers.

Therefore, the ALPS index reflects the ratio of water diffusivity along the peri-venous space (x-axis, Dx_projc and Dx_assoc) to diffusivity along other non-fiber-running directions (y-axis for projection fiber, Dy_proj; z for association fiber, Dz_assoc). As the index is dependent on ROI location, we took several methods to ensure they were correctly positioned. Firstly, based on observations, four ROIs were created using standard coordinates in the MNI space. Specifically, a 3D cross-hair containing 7 voxels (56 mm^3^) was placed at each voxel coordinates [25 52 50], [31 52 50], [64 52 50], [58 52 50] to locate left association, left projection, right association and right projection fiber (Figure 1) at the level of lateral ventricle body. By applying image normalization and using standard MNI coordinates, biases during ROI placement could be largely reduced. In most subjects the ROIs overlapped well with the target fiber tracts. Secondly, one observer visually checked the location of ROIs. Specifically, the eigenvector maps were visualized with color-coding to show the orientation of fiber tracts and the ROIs were overlaid in fsleyes. The observer checked whether the ROIs were fully located within the target fiber tract and manually adjusted their positions. Usually the adjustments were small, on the scale of shifting one or two voxels. Thirdly, reconstructed SWI (see below) images were also co-registered to DTI images, and the same observer checked the spatial consistency between ROIs and DMVs.

In the original study, the authors only measured ALPS in the left hemisphere as all subjects were right-handed and the fiber tracts are thick enough to place ROIs on the left side. Here we found that after image normalization, the fiber tracts were thick enough for bilateral measurement. Therefore, we used the mean ALPS from both hemispheres. Nevertheless, we also performed validation analysis using ALPS from each hemisphere. Additionally, to exclude possible influence of WMH on ALPS, we co-registered FLAIR and diffusion images and calculated the overlapping volume between WMH masks and ALPS ROIs (WMH_overlap_). The WMH_overlap_ was then used as a covariate during statistical analyses.

#### Brain Volume Analysis

The estimation of brain volume was performed using CAT12^3^. The T1 structural images were subjected to preprocessing steps for the correction of magnetic inhomogeneity, skull strip, and tissue segmentations. The intracranial volume (ICV) was calculated as the sum of white matter, gray matter and CSF (Malone et al., 2015). Brain tissue volume was calculated as the sum of gray and white matter. The Brain-to-ICV ratio was used to reflect brain atrophy.

#### Assessment of CSVD Markers

As described in our previous study (Huang et al., 2021a), PVS dilation, lacunes and microbleeds were visually assessed according to the STandards for ReportIng Vascular changes on nEuroimaging (STRIVE) by an experienced neuroradiologist (RZ). Briefly, PVS was assessed in both basal ganglia (BG) and deep white matter regions (WM), based on reconstructed thick-slice (5 mm) T2 images. Scores were given to dilated PVS in the basal ganglia and centrum semiovale as follows: 0 = no PVS, 1 = 10 PVS, 2 = 10–20 PVS, 3 = 21–40 PVS, and 4 = > 40 PVS (Potter et al., 2015). Lacunes were defined as a round or ovoid, subcortical, fluid-filled cavity (signal similar to CSF) with diameters ranging from 3 to 15 mm, consistent with a previous acute small subcortical infarct or hemorrhage in the territory of one perforating arteriole. Microbleeds were defined as small areas of signal void with associated blooming seen on susceptibility-weighted MRI. White matter hyperintensity (WMH) segmentation was performed using BIANCA^4^, and manually corrected by an experienced radiologist. The total lesion volume normalized by ICV was used to reflect the severity of WMH.

#### Measurement of DMVs

To generate optimal vessel-tissue contrast for DMVs, we adopted a two-stage phase unwrapping procedure (Ye et al., 2019) for SWI image reconstruction (Haacke et al., 2015), to fully preserve the high spatial frequency information for small vessels visualization and to minimize remnant susceptibility artifacts. The SWI images of all echoes were then averaged (Denk and Rauscher, 2010) to generate the final SWI images with improved SNR.

**FIGURE 2:**
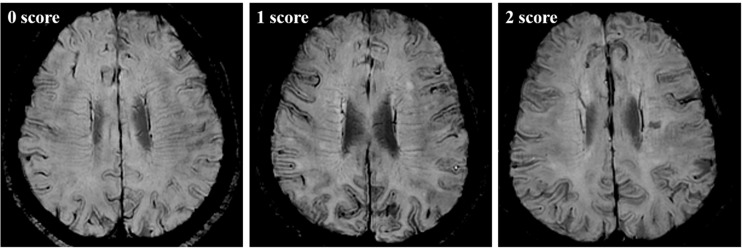
Example images of venous disruption on SWI images.

As described in our previous study, We assessed DMVs on reconstructed SWI images from the level of the lateral ventricles first appeared (top level) to the level of caudate first appeared (bottom level), which were about five consecutive periventricular slices (10 mm thick), considering these slices cover a large portion of DMVs (Zhang et al., 2017). We used a 3-point score to evaluate the DMVs (Figure 2): 0 score—each vein was continuous and had homogeneous signal; 1 score—each vein was continuous, but one or more than one vein had inhomogeneous signal; 2 score—one or more veins were not continuous. In this study, we had not divided DMV subregions, and the DMV scores were given according to the DMV integrity in all brain regions bilaterally, including frontal, parietal and occipital regions. Two neuroradiologists (YZ and RZ), who were blinded to other clinical and imaging data of the patients performed the assessments independently. The Cohen’s kappa was used to measure the consistency between the two observers.

### Statistical Analysis

First, univariate analyses were performed to investigate the relationship between ALPS and each predictor. Pearson’s correlation was used for revealing the association between ALPS and continuous variables. Two-sample *t*-test was used for categorical variables (sex, vascular risk factors) and one-way analysis of variance (ANOVA) was used for venous score. Second, we performed multiple regression analysis to understand the independent contribution of each factor to ALPS. Specifically, the ALPS index was set as dependent variable. Age, sex, hypertension, hyperlipemia, diabetes, smoking, PSQI, ICV, Brain-to-ICV ratio, normalized WMH volume, bgPVS score, wmPVS score, number of lacunes, number of microbleeds, DMV score and WMH_overlap_ were all entered as independent variables. Forward and backward selection were performed to select the most predictive factors from which a linear model was built. To avoid biased fitting, multi-collinearity analysis and residual analysis were performed. Standardized beta was used to reflect the predictive power of each variable.

## Results

A total of 142 community subjects were included (mean age = 60.8, female/male = 79/63, Table 1). Among all subjects, 56 (39.4%) had hypertension, 23 (16.2%) had hyperlipemia, 14 (9.99%) had diabetes and 40 (28.2%) were current or former smokers. In general, they had mild brain vascular burden. The median of WMH volume was 1.5 ml. Eleven of them had lacunes and 15 of them had microbleeds.

**TABLE 1:** Characteristics of the subjects.

Characteristics	*N* = 142
Age, y, mean ± SD	60.8 ± 7.0
Female, n (%)	79 (55.6%)
Education, y, mean ± SD	7.69 ± 3.7
Hypertension, n (%)	56 (39.4%)
Hyperlipemia, n (%)	23 (16.2%)
Diabetes, n (%)	14 (9.99%)
Smoker, n (%)	40 (28.2%)
MMSE, mean ± SD	27.3 ± 3.3
MoCA, mean ± SD	21.9 ± 4.8
PSQI, mean ± SD	5.4 ± 3.9
bgPVS score, median (interquartile range)	1 (1∼1)
wmPVS score, median (interquartile range)	1.5 (1∼2)
WMH volume, mL, median (interquartile range)	1.5 (0.8∼2.7)
Lacune, n, median (interquartile range)	0 (0∼0)
Microbleed, n, median (interquartile range)	0 (0∼0)

*PVS, perivascular space; WMH, white matter hyperintensity.*

**FIGURE 3:**
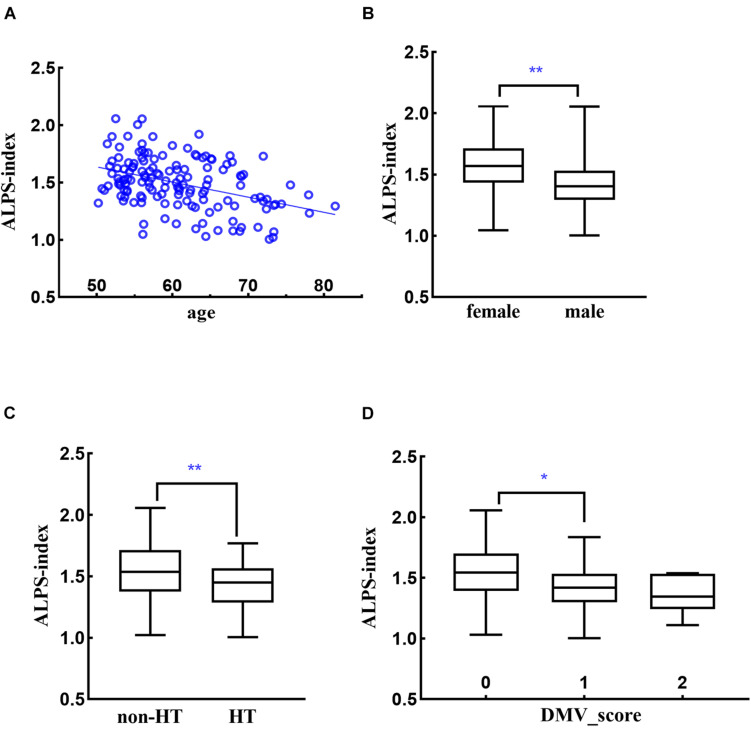
Relationships between ALPS-index and age **(A)**, sex **(B)**, hypertension [HT, **(C)**], and DMV score **(D)**. *p < 0.05, **p < 0.001.

The two observers had good agreement with each other (kappa = 0.643) on DMV assessment. Age was significantly associated with decreased ALPS (*r* = −0.416, *p* < 0.001) (Figure 3A). Compared to females, males had lower ALPS (1.56 vs. 1.41, *p* < 0.001) (Figure 3B). ICV had negative association with ALPS (*r* = −0.209, *p* = 0.012), while brain/ICV ratio was positively associated with ALPS (*r* = 0.397, *p* < 0.001). Hypertensive subjects had lower ALPS compared to non-hypertensive subjects (1.41 vs. 1.56, *p* < 0.001) (Figure 3C). Subjects with venous disruption had lower ALPS (venous score 0: 1.54, 1: 1.40, 2: 1.35, *p* < 0.001), and *post hoc* analysis showed that the difference came from the comparison of DMV score 0 vs. 1 (*p* = 0.001, Bonferroni correction) (Figure 3D). PSQI and WMH_overlap_ were not associated with ALPS. Normalized WMH volume, bgPVS score, number of lacunes and number of microbleeds were associated with decreased ALPS (*r* = −0.272, *p* = 0.001; *r* = −0.184, *p* = 0.028; *r* = −0.203, *p* = 0.015; *r* = −0.197, *p* = 0.018).

In multiple regression analysis, the variance inflation factors showed low multicollinearity among variables. Age, sex, hypertension, and venous score were selected in the final model (adjusted *R*^2^ = 0.319, Table 2, Model 1). The strongest predictor was age (β = −0.284, *p* < 0.001), followed by sex (β = −0.243, *p* = 0.001), DMV score (β = −0.215, *p* = 0.003) and hypertension (β = −0.189, *p* = 0.011). The CSVD imaging markers were no longer associated with ALPS in multiple regression analysis. Validation analyses using ALPS from each hemisphere also showed similar results (Table 2, Model 2 and 3).

**TABLE 2:** Regression analyses results.

Variables	Std beta	*P*-value
**Model 1, mean ALPS, *R*^2^ = 0.319**		
Age	−0.284	<0.001
Sex	−0.243	0.001
Hypertension	−0.189	0.011
DMV score	−0.215	0.003
**Model 2, left ALPS, *R*^2^ = 0.211**		
Age	−0.216	0.007
Sex	−0.258	0.001
Hypertension	−0.087	0.272
DMV score	−0.200	0.010
**Model 3, right ALPS, *R*^2^ = 0.129**		
Age	−0.206	0.014
Sex	−0.198	0.016
Hypertension	−0.154	0.065
DMV score	−0.025	0.757

## Discussion

In this study, we investigated the association between ALPS index and a variety of demographic factors, vascular risk factors and CSVD imaging signs. In general, we found results corresponded well with previous perceptions and results from animal studies, suggesting that glymphatic function was associated with a variety of demographic and vascular factors. The ALPS index might be a practical in-vivo method for measuring glymphatic function, but several factors need to be taken into consideration in future studies.

First, ALPS was negatively associated with age, and was lower in males. Glymphatic function drops dramatically during aging due to various factors. Reduced polarized aquaporin-4 channels on the endfeet of astrocyte cells (Valenza et al., 2019), decreased CSF production (Liu et al., 2020) and CSF pressure, diminished arterial pulsatility (Mestre et al., 2018b) could all lead to compromised glymphatic function in aged animals (Jessen et al., 2015). In a recent study, Zhou Y. et al. (2020). studied glymphatic function in human subjects using contrast enhanced imaging and showed that the speed of glymphatic clearance was related to aging. While the original ALPS research had not measured the correlation between ALPS and age, another follow-up study demonstrated negative correlations (Zhou W. et al., 2020).

The influence of sex on glymphatic function is still under investigation. Two recent animal studies derived seemingly different results. Giannetto et al. (2020) found that there was no sex difference in total influx or subregion-dependent tracer distribution in mice at different ages, while Liu’ study (Liu et al., 2020) found elevated CSF production in female mice. Human imaging study also showed no difference of glymphatic clearance between males and females. The difference might be related to CSF transport kinetics. Males usually have larger artery diameter and more PVS dilation compared to females (Zhang et al., 2014). On the other hand, females tend to have higher blood speed in common carotid arteries (Azran et al., 2010). The combination of these factors may cancel each other’s effect and yield comparable glymphatic clearance. As the ALPS index indirectly reflects the speed of fluid motion in peri-venous space, the water diffusivity may indeed be higher in females.

Second, hypertensive subjects had significantly lower ALPS compared to non-hypertensive subjects. The small vessel diseases including hypertensive arteriosclerosis are speculated to have the aspect of “interstitial fluidopathy,” which is the concept of diseases whose pathologies are associated with abnormal interstitial fluid dynamics (Taoka and Naganawa, 2021). Studies have proven that arterial pulsatility is an important driving force of CSF flow, which might significantly decrease in hypertensive condition (Mestre et al., 2018b). Therefore, hypertensive subjects have decreased glymphatic function (Zhou Y. et al., 2020). and are at greater risk of neurodegenerative diseases (Jessen et al., 2015). While we evaluated CSF/ISF dynamics at the venous side, the effect of hypertension could still be reliably detected.

Third, ALPS was lower in subjects with venous disruption. While signals from blood within the DMVs were supposed to be decayed using diffusion imaging with *b* = 1,000, venous disruption might have significant influence on the index. During brain aging, DMVs tend to become tortuous due to blood pressure changes and collagenosis-related stenosis (Keith et al., 2017). Several studies using 7T-MR susceptibility imaging suggested that venular tortuosity could be an early marker of CSVD and Alzheimer’s disease (Bouvy et al., 2017; Shaaban et al., 2017). Under such conditions, PVS around DMVs may also be bended, making the fluid movement slower, especially on the left-right axis. This issue should be taken into consideration in future studies utilizing ALPS to measure glymphatic function *in vivo*.

Fourth, the bgPVS score was associated with decreased ALPS index in univariate analysis. PVS dilation has been found associated with many neurological disorders (Doubal et al., 2010; Chen et al., 2011; Francis et al., 2019). Previous studies suggest that PVS dilation may be attributed to amyloid deposition (Roher et al., 2003; Keable et al., 2016), BBB breakdown (Mestre et al., 2017), inflammation and brain atrophy (Wardlaw et al., 2020), etc. Accumulated amyloid plaques and inflammatory cell debris in PVS may hinder fluid movement and lead to decreased ALPS-index. Hydro-kinetic changes may also contribute to decreased glymphatic function, because pulsatility force could decrease when the diameter of the conduit increases. Nevertheless, the combined effect of all related factors is difficult to estimate. When putting together with vascular risk factors and other CSVD markers, PVS dilation was not independently associated with glymphatic function, which may suggest that they were both resulted from aging (Zhou Y. et al., 2020). and vascular related damages, but there was no causal relationship between them.

Fifth, in addition to the demographic and vascular risk factors analyzed in this study, there are other possible factors that may affect the ALPS index. Respiratory and arterial pulsation related CSF flow might contribute to the flushing in PVS (Daversin-Catty et al., 2020; Wardlaw et al., 2020), and may change from awakening to sleep. The definition of ROIs, including their locations and size, may determine the accuracy of measurement and should be well-controlled. With higher image resolution, localization could be more precise and thus the ALPS index would be more accurate. Genetic variation in the aquaporin-4 gene may also have significant influence on glymphatic drainage (Mestre et al., 2018a).

The current study has several limitations. Currently, the contrast-enhanced imaging methods were considered relatively reliable for measuring glymphatic clearance (Ringstad and Eide, 2020). However, it is quite difficulty to perform such experiments in healthy subjects, especially in a sample size as large as ours. As most of our results were in good agreement with previous studies, we still consider that the ALPS index could reflect glymphatic function and is a promising tool for measuring glymphatic function *in vivo*. Though, cautions should be taken regarding several confounding factors such as hypertension and venous disruption. Another weakness is the relatively low imaging resolution for SWI (in-plane resolution: 0.75 mm ^∗^ 0.75 mm). This limited our ability to observe subtle changes in DMV and we used a 3-point scale rather than the 18-point scale used in our previous studies. Nevertheless, we still could observe significant association between venous degeneration and ALPS, suggesting that their relationship was quite robust. Finally, the associations between ALPS and several glymphatic related factors (diabetes, sleep disorder) were not found in our study. This was possibly due to the low number of diabetic subjects, and that most subjects had relatively good sleep conditions. While a recent study (Yang et al., 2020) suggests that patients with type 2 diabetes mellitus had reduced ALPS, their sample size was also very small. Future studies should investigate the influence of these factors in more dedicated large samples.

## Data Availability Statement

The original contributions presented in the study are included in the article/supplementary material, further inquiries can be directed to the corresponding author/s.

## Ethics Statement

All procedures performed in studies involving human participants were in accordance with the ethical standards of the institutional research committee and with the 1964 Helsinki declaration and its later amendments or comparable ethical standards. All subjects signed informed consent before enrollment. The patients/participants provided their written informed consent to participate in this study. We certify that we have participated sufficiently in the work to take public responsibility for the appropriateness of the experimental design and method, and the collection, analysis, and interpretation of the data. All authors have read and approved the submitted manuscript, and the manuscript has not been submitted elsewhere nor published elsewhere in whole or in part.

## Author Contributions

PH and MZ were responsible for the study concept and design and provided critical revision of the manuscript for important intellectual content. RZ, YJ, SW, HH, KL, QZ, XL, XY, XX, XW, and PH contributed to the acquisition of imaging data. YZ and RZ performed data analysis, interpreted the findings, and drafted the manuscript. All authors critically reviewed the content and approved the final version for publication.

## Conflict of Interest

The authors declare that the research was conducted in the absence of any commercial or financial relationships that could be construed as a potential conflict of interest.

## Footnotes

1 https://fsl.fmrib.ox.ac.uk/fsl/fslwiki

2 www.mrtrix.org

3 http://www.neuro.uni-jena.de/cat/

4 https://fsl.fmrib.ox.ac.uk/fsl/fslwiki/BIANCA
